# The relationship between social risk factors and latent tuberculosis infection among individuals residing in England: a cross-sectional study

**DOI:** 10.1136/bmjgh-2020-003550

**Published:** 2020-12-07

**Authors:** Swaib A Lule, Rishi K Gupta, Maria Krutikov, Charlotte Jackson, Jo Southern, Ibrahim Abubakar

**Affiliations:** 1 Institute for Global Health, University College London, London, UK; 2 Tuberculosis (TB) Unit, National Infection Service, Public Health England, London, UK

**Keywords:** epidemiology, public health, tuberculosis

## Abstract

**Objective:**

To investigate the relationship between social risk factors and latent tuberculosis infection (LTBI) among individuals who are eligible for LTBI screening in the United Kingdom (UK).

**Methods:**

This cross-sectional study used data collected in the UK Prognostic Evaluation of Diagnostic Interferon-Gamma Release Assays (IGRAs) Consortium Study which enrolled 9176 recent tuberculosis (TB) contacts and migrants at National Health Service (NHS) facilities and community settings in the UK. The study outcome was LTBI (positive IGRA test (QuantiFERON-TB Gold In-Tube or T-SPOT.TB)). The main exposures were history of smoking, history of substance misuse, homelessness, prison stay and socioeconomic deprivation.

**Results:**

4914 (56.2%) individuals resided in the most deprived areas and 2536 (27.6%) had LTBI. In the multivariable analysis (adjusting for age, gender, place of birth, ethnicity, HIV status, BCG vaccination and recent TB contact) living in the least deprived areas compared with living in the most deprived areas was associated with reduced odds of LTBI (odds ratio (OR)=0.68, 95% CI: 0.51 to 0.90) while ever been homeless (OR=1.50, 95% CI: 1.02 to 2.21) was associated with increased odds of LTBI. Smoking, homelessness and substance misuse were not associated with LTBI.

**Conclusion:**

Social deprivation could be an important risk factor for LTBI, highlighting the social inequality in the burden of TB infection in the UK. Migrants and TB contacts who were socially deprived or homeless were at a significantly higher risk for LTBI, thus tailored intense public health interventions to these groups may help to reduce the risk of future TB disease.

**Trial registration number:**

ClinicalTrials.gov Registry (NCT01162265).

**Key questions**

**What is already known?**

- Undiagnosed and untreated LTBI is an important reservoir for future or re-emerging TB disease.
- Global strategies and national guidance emphasise screening and treatment of LTBI as a method to prevent new TB cases.
- It is not clear if social risk factors and/or socioeconomic deprivation are independently associated with LTBI among individuals at high risk for TB, and to what extent any such association may be explained by recent TB contact, ethnicity and/or place of birth.

**What are the new findings?**

- Social deprivation and homelessness could be important drivers for LTBI among migrants to the UK, and or contacts of people with TB in the UK.

**What do the new findings imply?**

- Targeting LTBI screening programmes towards people at high risk of TB who are socially deprived and or homeless may increase the yield of LTBI screening and positively impact on TB control efforts in the UK.

## Introduction

Despite declining tuberculosis (TB) incidence in most European countries such as the United Kingdom (UK), large numbers of individuals continue to suffer from this preventable disease.^1^ Undiagnosed and untreated latent TB infection (LTBI) remains an important reservoir for emerging TB disease.^2^ Most TB cases are thought to occur as a result of reactivation of latent infection.^3 4^ If untreated, each person with active TB may infect on average between 10 and 15 people each year. Globally, TB remains an important public health problem causing more than a million deaths annually.^5^

Global strategies and national guidance emphasise screening and treatment of LTBI as a method to prevent new TB cases.^1 6 7^ Preventing new infections and/or treating existing LTBI can interrupt TB transmission,^8 9^ hence targeted LTBI screening and treatment in high-risk groups is a vital intervention for effective TB control.^10 11^

In 2018, the annual TB incidence in the UK was 9.3 per 100 000 people.^6^ The majority of TB cases in England occurred in major cities and among non-UK-born individuals.^2 6^ Rates of TB were highest among individuals from the most deprived areas compared with those from the least deprived areas,^3 6^ for example in 2018, the rates of TB were 16.6 per 100 000 in the most deprived 10% of the population compared with 3.0 per 100 000 in the least deprived 10%.^6^

In the 1980s, measures of deprivation including being a migrant, residing in overcrowded housing and unemployment, were associated with higher rates of TB in the UK.^12 13^ Illicit drug use and prison stay were also associated with TB infection^14 15^ However, the relative role of factors such as socioeconomic deprivation as a driver of LTBI in settings with high proportions of resident migrants is less clear, although it is known that rates of TB disease are highest in the poorest areas of the UK.^3 6^

Historically, contacts of patients with TB have been offered LTBI testing and treatment based on national guidelines. In 2016, LTBI screening and treatment was implemented across England, targeting recent migrants into the UK from high TB-burden countries,^3 6^ though to date, there is no such programme for individuals with social risk factors for TB disease.

We used data from the UK Prognostic Evaluation of Diagnostic Interferon-Gamma Release Assays (IGRAs) Consortium (PREDICT) study to investigate the association between social risk factors and LTBI among individuals at risk of TB infection, which we defined as social deprivation, smoking, homelessness, prison stay and drug use. Understanding the role of social drivers for LTBI among migrants and individuals recently exposed to TB could ensure that these groups are prioritised in the delivery of existing interventions against LTBI.

## Methods

### Study design and participants

This analysis used baseline data on participants in the PREDICT prospective cohort study, which was designed to assess the prognostic ability of the two commercially available IGRAs and the standard Mantoux tuberculin skin test (TST) in predicting active TB among untreated individuals with LTBI.^16 17^ Individuals were eligible for inclusion in PREDICT if they were a recent contact of an individual with active TB, or were a new entrant into the UK from a high TB-burden country (or frequently visited these countries).^16 17^

Recruitment took place between 4th of May 2010 and 1st of June 2015 at National Health Service (NHS) centres (including tuberculosis clinics, and general practices) and community settings (places of worship [like Hindu and Sikh temples, mosques and churches] schools and colleges, and workplaces) in London, Birmingham and Leicester.^16 17^. Written informed consent was obtained from each individual for their participation. No member of the public or study participant was involved in the design, or conduct, or reporting or dissemination plans of the research.

### Data collection and assessment of latent tuberculosis

Trained research nurses interviewed participants and collected demographic and socioeconomic data using standardised paper questionnaires.^16^ Participants were tested for LTBI using the Mantoux TST and two IGRAs: QuantiFERON TB Gold In-Tube test (QFT-GIT; Qiagen, Hilden, Germany) and the T-SPOT.TB (Oxford Immunotec, Oxford, UK).^16 17^ Results were classified as recommended by the manufacturers for IGRA and in accordance with the National Institute for Clinical Excellence for TST.^4^

### Measure of socioeconomic deprivation

Social deprivation is the extent to which a person, or a community, lacks what they need to have a decent life, such as education, housing, employment and healthcare.^18^ Area socioeconomic deprivation was measured using the Index of Multiple Deprivation (IMD)-2015 linked to participants’ postcodes at the time of recruitment. The IMDs were generated by the Office for National Statistics as a measure of relative deprivation for small areas or neighbourhoods in England. The IMD-2015 relates to the 2012/2013 tax year which was midway through the study recruitment period. The IMD-2015 was based on 37 separate indicators from 7 distinct domains of deprivation including income, employment, education, health, crime, access to housing and services, and living environment combined to form the overall measure of multiple deprivation.^19 20^ Participants’ postcodes were entered in a ‘postcode look-up’ tool,^21^ to generate IMD (ranked from 1 (most deprived area) to 32 844 (least deprived area)) and the deciles of deprivation (from 1 (most deprived) to 10 (least deprived)) corresponding to small areas or neighbourhoods.^19 20^

### Study outcome, exposures and potential confounders

The primary outcome was LTBI defined as a positive result to either QFT-GIT or T-SPOT.TB test. The main exposures were social deprivation, smoking history (ever vs never), history of substance misuse (present vs absent), homelessness (present vs absent) and prison stay (present vs absent). Social deprivation was categorised using deciles of deprivation into the most deprived (deciles: 1–3), moderately deprived (deciles: 4–7) and least deprived (deciles: 8–10) areas. The other social risk factors (history of smoking, history of substance misuse, homelessness or prison stay) were self-reported. Social risk factors available in the PREDICT Study were included in the data analysis.

Potential confounders identified *apriori* were place of birth (UK or elsewhere), age in years treated as a continuous variable, gender (male or female), ethnicity (Asian, White, Black or Mixed/other), recent TB contact (yes or no), BCG vaccination status (yes or no) and HIV status (self-reported as negative or positive). BCG vaccination status was assessed based on the presence of a scar, recall or vaccination record.

### Statistical analysis

Data were analysed using Stata 14.2 (College Station, Texas, USA). The chi-squared (Χ^2^) tests were used to compare descriptive characteristics and socioeconomic factors between the PREDICT study participants who were included and not included in this study, and to assess the relationship between participants’ characteristics and the social risk factors. Univariable and multivariable logistic regressions were used to assess the association between each social risk factor and LTBI, adjusting for confounders to compute odds ratios (ORs and their 95% CI.

A multivariable logistic regression model was fitted to include LTBI (the main outcome), all the social risk factors (the main exposures) and all variables associated with either LTBI or any of the social risk factors at a p-value of <0.05 (confounders). Participants’ age was included in the final model *apriori*. The change in standard error (SE) method was used to assess multicollinearity between variables included in the final model. A sensitivity analysis assessing the linear associations between IMD (measured as both a continuous variable and as deciles of social deprivation) and the outcome of LTBI was done. All analyses used a complete case approach.

## Results

**Figure 1:**
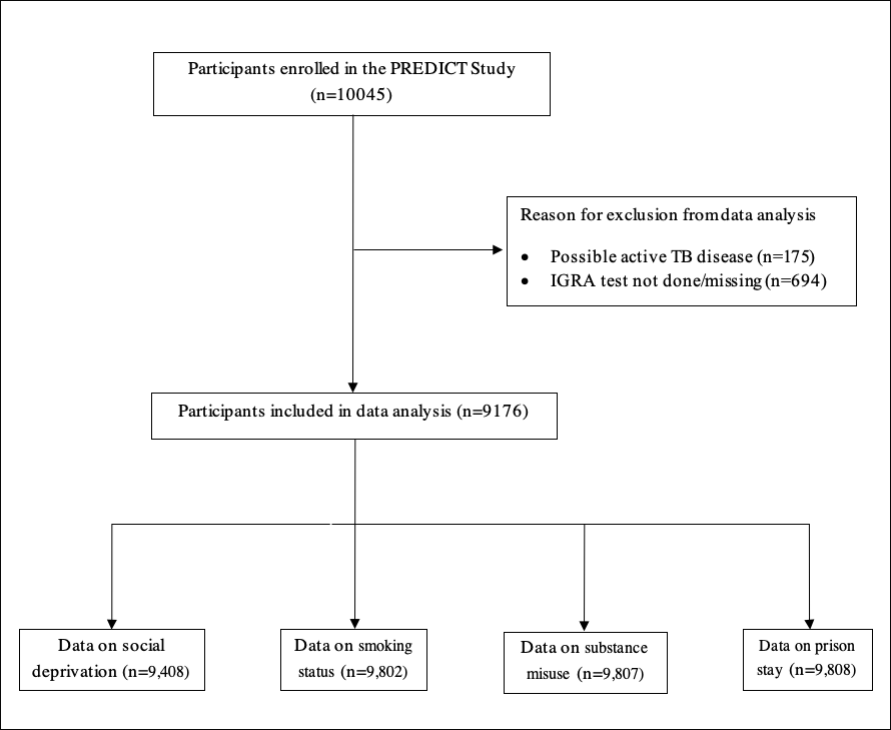
Flow chart of participants included in the data analysis. IGRA, Interferon-Gamma Release Assay; PREDICT, Prognostic Evaluation of Diagnostic IGRAs Consortium; TB, tuberculosis.

From 54 National Health Service (NHS) primary and secondary care facilities and various community settings in London, Birmingham and Leicester, 10 045 individuals were initially recruited into the PREDICT Study.^16^ Of these, 175 (1.7%) individuals were excluded due to possible active TB disease, while 9870 (98.3%) were considered for inclusion (figure 1). Of those eligible, 9176 (93.0%) individuals with available results for at least one IGRA test were included in this study. Overall, participants excluded were similar in characteristics to those included in the study, except they were more likely to have had recent TB contact. Details are shown in table 1.

**Table 1:** Characteristics of the PREDICT participants included and not included in the data analysis (N=9870)

Characteristic	Included (N=9176)		Not included (N=694)		P value*
	Number	Percentage	Number	Percentage	
Latent tuberculosis infection (N=9176)					
Positive	2536	27.6			
Area deprivation (N=9408)					
Most deprived	4914	56.2	335	51.0	
Moderately deprived	3355	38.3	278	42.3	
Least deprived	482	5.5	44	6.7	0.031
Ever smoked (N=9802)					
Yes	1735	19.0	119	17.6	0.377
History of substance misuse (N=9807)					
Yes	193	2.1	11	1.6	0.401
Ever been homeless (N=9814)					
Yes	179	2.0	12	1.8	0.743
Ever been in prison (N=9808)					
Yes	126	1.4	12	1.8	0.394
Gender (N=9797)					
Female	4558	50.0	367	53.9	0.050
Age (N=9848)					
≤35 years	5404	59.0	382	55.8	
>35 years	3759	41.0	303	44.2	0.100
Place of birth (N=9820)					
Non-UK	7667	83.9	564	82.5	
UK	1469	16.1	120	17.5	0.316
Ethnicity (N=9618)					
Asian	5332	59.7	402	59.1	
White	1113	12.5	84	12.4	
Black	1310	14.7	121	17.8	
Mixed/other	1183	13.2	73	10.7	0.065
HIV status(self-reported)(N=9183)					
Positive	52	0.6	3	0.5	0.650
BCG vaccination (N=8312)					
Yes	6346	81.8	450	81.7	0.954
Recent TB contact (N=9768)					
Yes	4605	50.7	412	59.8	<0.001

Percentages may not add to 100 due to rounding.

*Χ^2^ test p value.

PREDICT, Prognostic Evaluation of Diagnostic Interferon-Gamma Release Assays Consortium; TB, tuberculosis.

Study participants had a median age of 32 years (interquartile range : 26–46); 4558 (50.0%) were women; 1469 (16.1%) were born in the UK; 5332 (59.7%) were of Asian ethnicity; 6346 (81.8%) had a previous BCG vaccination; 4914 (56.2%) resided in the most deprived areas and 2536 (27.6%) had LTBI. Other socioeconomic risk factors were uncommon in this study population, with 126 (1.4%) ever been in prison; 179 (2.0 %) ever been homeless; 1735 (19.0%) ever smoked and 193 (2.1%) had a history of substance misuse. Online supplemental table 1 shows the distribution of the IMD deciles for the 9176 individuals included in the analysis.

10.1136/bmjgh-2020-003550.supp1 Supplementary data

The association between participants’ characteristics and LTBI is shown in table 2. Briefly, there was evidence that social deprivation and homelessness were associated with LTBI on univariate analysis. Other factors associated with LTBI on univariate analysis were gender, age, place of birth, ethnicity and recent TB contact. The crude association between each social risk factor and LTBI from the logistic regression showed social deprivation and homelessness were both associated with LTBI (table 2). Compared with individuals residing in the most deprived areas, those in the moderately deprived areas had 4% lower odds of LTBI (unadjusted OR=0.96, 95% CI: 0.87 to 1.06), while individuals in the least deprived areas had a 32% reduction in odds of LTBI, (unadjusted OR=0.68, 95% CI: 0.54 to 0.86). Individuals who reported having ever been homeless had increased odds of LTBI compared with individuals who had never been homeless with unadjusted OR=1.54, 95% CI: 1.13 to 2.10. There was no evidence that other social risk factors (smoking, history of substance misuse or prison stay) were crudely associated with LTBI (table 2). Online supplemental tables 2 and 3 show the crude relationship between participants’ characteristics and the social risk factors and the relationship between social risk factors.

**Table 2:** Relationships between participant characteristics and latent tuberculosis infection (LTBI) among individuals in the UK

Characteristics	LTBI					
	Negative N (%)	Positive N (%)	Crude OR (95% CI)	P value	Adjusted OR (95% CI)	P value
Area deprivation						
Most deprived	3527 (71.8)	1387 (28.2)	1		1	
Moderately deprived	2436 (72.6)	919 (27.4)	0.96 (0.87 to 1.06)		0.92 (0.81 to 1.03)	
Least deprived	380 (78.8)	102 (21.2)	0.68 (0.54 to 0.86)	0.003	0.68 (0.51 to 0.90)	0.013
Ever smoked						
No	5356 (72.5)	2036 (27.5)	1		1	
Yes	1246 (71.8)	489 (28.2)	1.03 (0.92 to 1.16)	0.591	0.99 (0.85 to 1.15)	0.893
History of substance misuse						
No	6459 (72.2)	2482 (27.8)	1		1	
Yes	148 (76.7)	45 (23.3)	0.79 (0.56 to 1.11)	0.173	0.83 (0.55 to 1.25)	0.360
Ever been homeless						
No	6498 (72.5)	2462 (27.5)	1		1	
Yes	113 (63.1)	66 (36.9)	1.54 (1.13 to 2.10)	0.006	1.50 (1.02 to 2.21)	0.043
Ever been in prison						
No	6524 (72.4)	2484 (27.6)	1		1	
Yes	83 (65.9)	43 (34.1)	1.36 (0.94 to 1.97)	0.104	1.35 (0.85 to 2.15)	0.210
Gender						
Male	3152 (69.2)	1406 (30.9)	1		1	
Female	3443 (75.5)	1115 (24.5)	0.73 (0.66 to 0.80)	<0.001	0.69 (0.62 to 0.77)	<0.001
Age (years)			1.01 (1.00 to 1.01)	<0.001	1.01 (1.00 to 1.01)	<0.001
Place of birth						
Non-UK	5391 (70.3)	2276 (29.7)	1		1	
UK	1223 (83.3)	246 (16.8)	0.41 (0.41 to 0.55)	<0.001	0.41 (0.34 to 0.49)	<0.001
Ethnicity						
Asian	3895 (73.1)	1437 (27.0)	1		1	
White	879 (79.0)	234 (21.0)	0.72 (0.62 to 0.84)		0.87 (0.71 to 1.07)	
Black	870 (66.4)	440 (33.6)	1.37 (1.20 to 1.56)		1.29 (1.10 to 1.52)	
Mixed/other	835 (70.6)	348 (29.4)	1.13 (0.98 to 1.30)	<0.001	1.11 (0.93 to 1.31)	0.002
HIV status						
Negative	6125 (72.2)	2362 (27.8)	1		1	
Positive	38 (73.1)	14 (26.9)	0.96 (0.52 to 1.77)	0.884	0.80 (0.40 to 1.62)	0.535
BCG vaccination						
No	1025 (72.4)	390 (27.6)	1		1	
Yes	4620 (72.8)	1726 (27.2)	0.98 (0.86 to 1.12)	0.781	0.89 (0.78 to 1.03)	0.128
Recent TB contact						
No	3333 (74.5)	1141 (25.5)	1		1	
Yes	3231 (70.3)	1374 (29.8)	1.24 (1.13 to 1.36)	<0.001	1.44 (1.27 to 1.63)	<0.001

Percentages may not add to 100 due to rounding.

A total of 6488 individuals included in the multivariable analysis.

Multivariable logistic regression model was fitted to include LTBI, all of the social risk factors and all variables associated with LTBI or one of the social risk factors.

The final model included LTBI, the social risk factors (deprivation, smoking, substance misuse, homelessness or prison stay) and the potential confounders (age, gender, ethnicity, recent TB contact, prior BCG vaccination, place of birth). In multivariable analyses (n=6488) social deprivation and homelessness were associated with LTBI. The adjusted ORs for LTBI among individuals in moderately and least deprived areas were 0.92, 95% CI (0.81 to 1.03) and 0.68, 95% CI (0.51 to 0.90), respectively, compared with those residing in the most deprived areas. After adjustment for confounders, individuals who reported ever been homeless were 1.5 times more likely to have LTBI (OR=1.50, 95% CI: 1.02 to 2.21) compared with individuals who had never been homeless. There was no evidence that the other social risk factors were associated with LTBI at multivariable analysis (table 2).

There was no multicollinearity between variables included in the final model. The IMD-2015 measured access to housing and crime which potentially relate to homelessness and prison stay, respectively. The effect of both prison stay and homelessness on LTBI was not affected by social deprivation in the final model, for example, the ORs for homelessness and prison stay were 1.45, 95% CI (1.00 to 2.11) and 1.40, 95% CI (0.89 to 2.19), respectively, on dropping social deprivation from the final model.

Results from sensitivity analyses were consistent with the main finding; social deprivation (as IMD or deciles of social deprivation) was associated with LTBI. For example, the unadjusted odds of LTBI decreased by 4% for every increase in the deprivation decile, OR=0.96, 95% CI (0.94 to 0.98). At multivariable analysis, decreasing level of deprivation was weakly associated with a reduction in LTBI: a one unit increase in deprivation decile was associated with a 4% reduction in odds of LTBI, OR=0.96, 95% CI (0.93 to 0.99).

## Discussion

This cross-sectional study has shown that increasing social deprivation and homelessness were associated with LTBI in a population with high TB risk, where LTBI was common. Other social risk factors (smoking, substance misuse, or prison stay) were not associated with LTBI in this population. Although smoking was strongly associated with social deprivation, it was not independently associated with LTBI in this population.

The prevalence of LTBI at 27.6% in this study was higher than the LTBI prevalence for England (at 15.8%) in 2018, probably due to the study locations being large cities^6^ and the intentional recruitment of participants at high risk of LTBI. In England, the percentage of positive test for LTBI has gradually dropped over the years, for example from 18.1% in 2016 to 17.0% in 2017 and 15.8% in 2018.^6^ Except for social deprivation and homelessness, this study found no association between other social risk factors (smoking, substance misuse and prison stay) and LTBI. In contrast, a study at admission into UK remand prisons showed that LTBI was associated with substance misuse.^22^ Studies from Asia have also shown associations between social risk factors (such as smoking, substance misuse, homelessness and prison stay) and LTBI.^23 24^

The increased risk of LTBI in most deprived areas could be attributed to several factors such as overcrowded households and settlements, or limited access to healthcare especially among very new and/or undocumented migrants. In the UK, undocumented/illegal migrants (especially those not identified through the pre-entry screening programme for migrants from high TB-burden countries) may not only lack income but have limited access to social services such as housing facilities and healthcare, limiting their opportunities for treatment of either LTBI or TB disease and thus facilitating onward transmission. Therefore, prioritising LTBI screening among socially deprived and or homeless individuals at risk of TB exposure could impact TB control by ensuring that individuals at the highest risk of LTBI are treated, so progression to active TB and subsequent TB transmission is interrupted.

Although there was no multicollinearity, the social risk factors included in the analysis may, to a certain extent, be similar to one another. For example, ecological data on homelessness (one of the deprivation indicator used to generate the IMD-2015) may be related to individual-level data on homelessness, one of the social risk factors analysed in the current study. The absence of association between these social risk factors (smoking, substance misuse and prison stay) and LTBI may partly be due to the similarity of these variables in indicating social deprivation, as well as to the small numbers of participants reporting these social risk factors. In this study, individuals who reported homelessness were more likely to report having ever spent time in a prison. Individuals in the same IMD may be more similar or have similar level of exposure. Overall, there were 3.6 individuals per IMD who may be similar in characteristics to others in the same IMD.

The World Health Organization guidelines call for LTBI testing in high risk and vulnerable groups including migrants, individuals experiencing homelessness, those in correctional facilities and residents of long-term care facilities.^7^ Previous research supports the notion of ‘knowing your epidemic’ by using surveillance data to tailor a response.^25^ Our study supports this approach by showing a higher prevalence of LTBI in subgroups of the population that may benefit from further targeted interventions. Studies prospectively collecting data on important social deprivation and or study the impact each of the domains of IMD are required to further examine relationship between social risk factors and LTBI. Also, further studies investigating whether it is cost-effective to prioritise LTBI screening (for example, during contact tracing) by socioeconomic groups are needed to inform TB control, especially in low-burden countries.

In this study, foreign-born individuals were more likely to reside in the most deprived areas than UK-born individuals. Implementation of LTBI screening services in most deprived areas may be challenging and hampered by limited uptake and acceptance due to structural issues that prevent access, including rights to and knowledge of free diagnosis.^26^ In 2010, the UK introduced measures to make access to public services more difficult for undocumented migrants, as a consequence, there are fears by undocumented migrants that migration regulation could be enforced when they access health services.^27^ For effective LTBI screening programmes in the most deprived areas, the UK government should expand and enhance health systems, to incorporate the needs of undocumented migrants. The use of a firewall between immigration authorities and the NHS screening system and the provision of free treatment may enhance engagement.^28^

### Study strengths, limitations and mitigations

The IGRAs are the most specific measure of LTBI currently available, and each IGRA test was conducted independent of the other, based on manufacturer-defined standardised methods.

As the PREDICT study did not obtain data to allow the accurate assessment of individual deprivation levels, we used an area measure of deprivation. Earlier studies have shown relationships between IMD and various health inequalities and mortality^29 30^ and the consistency of our results with other proxy measures such as homelessness supports a true association. Further limitations include the possibility of residual confounding by unmeasured factors (such as individual-level educational or employment status or social deprivation premigration, morbidity), the cross-sectional nature of the data as we used baseline data from a cohort study for this analysis and important social risk factors, such as residing in overcrowded housing and unemployment, were missing. The exposure, outcome and potential confounders were assessed according to the same standards for all participants limiting any ability to understand temporality of the observed association. Among migrants, current deprivation might reflect previous deprivation before migrating to the UK.

Furthermore, data on social risk factors and HIV status were self-reported and may not have been reported accurately. For example, postcode (used to derive deprivation data) may not always be accurate as individuals may provide family addresses or postcodes of their previous residence. Deprived and homeless individuals are more likely to have missing data which may lead to a selection bias. Of the individuals included in this study, 6459 (70.4%) with complete data were included in the final model. The complete case analysis approach results in loss of information and may result in biased estimates, either as an underestimation or overestimation of the effect size. However, participants included in the analysis were similar in characteristics to those excluded, thus missing data were unlikely to introduce a bias in this study. Homeless individuals are likely to have had a postcode of the hospital or clinic they were recruited from resulting in non-differential misclassification of social deprivation and thus bias the estimate towards null.

### Generalisability

The findings of this study may not apply to the general UK population but are generalisable to populations with high TB risk in the UK. Study participants were sampled from areas of England, and among migrants and contacts where there is a higher LTBI and/or TB prevalence than the general UK population.

In conclusion, our study suggests that migrants and contacts with TB in deprived areas may be more likely to have LTBI, thus targeted intense public health interventions among those socially deprived could have an impact on the yield of LTBI screening and may help to reduce the impact of future TB disease in the UK. In the long term, interventions that address socioeconomic deprivation and inequalities may contribute to reducing the prevalence of LTBI in low TB-incidence countries with a potential impact on the overall TB burden.
