# Supplementary material for: The relationship between social risk factors and latent tuberculosis infection among individuals residing in England: a cross-sectional study
Source: BMJ Glob Health. 2020 Dec 7;5(12):e003550. doi: 10.1136/bmjgh-2020-003550 (PMC7722758; doi:10.1136/bmjgh-2020-003550)
Supplement: Supplementary data [file bmjgh-2020-003550supp001.pdf]

Table S1 Distribution of the index of multiple deprivation decile (N= 9176)

IMD decile	Number	Percentage
1 (Most deprived)	761	8.7
2	1595	18.2
3	2558	29.2
4	1451	16.6
5	904	10.3
6	630	7.2
7	370	4.2
8	267	3.1
9	125	1.4
10 (Least deprived)	90	1.0

IMD; index of multiple deprivation
Percentages may not add to 100 due to rounding
465 individuals had missing values of IMD

Table S2 Relationship between participant characteristic and each of following social factor (deprivation, substance misuse)

Characteristics	Area deprivation				History of substance misuse		
	Most	Moderately	Least	P-value	No	Yes	P-value
	N (%)	N (%)	N (%)		N (%)	N (%)	
Area deprivation							
Most-deprived					4795 (97.9)	102 (2.1)	
Moderately-deprived					3268 (97.7)	76 (2.3)	
Least-deprived					468 (98.1)	9 (1.9)	<0.778
Ever smoked							
No	3901 (55.3)	2749 (39.0)	401 (5.7)		7351 (99.6)	32 (0.4)	
Yes	991 (59.7)	591 (35.6)	79 (4.8)	0.005	1569 (90.8)	160 (9.3)	<0.001
History of substance misuse							
No	4795 (56.2)	3268 (38.3)	468 (5.5)				
Yes	102 (54.6)	76 (40.6)	9 (4.8)	0.778			
Ever been homeless							
No	4798 (5617)	3283 (38.4)	477 (5.6)		8779 (98.1)	169 (1.9)	
Yes	102 (61.8)	61 (37.0)	2 (1.2)	0.036	155 (86.6)	24 (13.4)	<0.001
Ever been in prison							
No	4820 (56.1)	3303 (38.4)	476 (5.5)		8835 (98.2)	163 (1.8)	
Yes	75 (62.5)	43 (35.8)	2 (1.7)	0.116	94 (75.8)	30 (24.2)	<0.001
Gender							
Male	2481 (56.9)	1653 (37.9)	230 (5.3)		4393 (96.7)	148 (3.3)	
Female	2409 (55.5)	1680 (38.7)	250 (5.8)	0.361	4499 (99.0)	44 (1.0)	<0.001
Place of birth							
Non- United Kingdom	4191 (57.2)	2797 (38.2)	338 (4.6)		7552 (98.8)	94 (1.2)	

United Kingdom	709 (50.7)	548 (39.2)	141 (10.1)	<0.001	1366 (93.3)	98 (6.7)	<0.001
Ethnicity							
Asian	2663 (52.3)	2162 (42.4)	271 (5.3)		5272 (99.2)	43 (0.8)	
White	535 (50.9)	385 (36.6)	132 (12.6)		1036 (93.3)	75 (6.8)	
Black	938 (75.0)	295 (23.6)	18 (1.4)		1275 (97.6)	31 (2.4)	
Mixed/Other	646 (57.0)	441 (39.9)	46 (4.1)	<0.001	1137 (96.6)	41 (3.5)	<0.001
HIV status							
Negative	4495 (55.5)	3147 (38.9)	457 (5.6)		8288 (97.8)	183 (2.2)	
Positive	30 (58.8)	20 (39.2)	1 (2.0)	0.516	48 (94.1)	3 (5.9)	0.070
BCG vaccination							
No	801 (59.1)	480 (35.4)	74 (5.5)		1372 (97.4)	37 (2.6)	
Yes	3366 (55.6)	2357 (38.9)	331 (5.5)	0.050	6205 (97.9)	132 (2.1)	0.207
Recent TB contact							
No	2490 (58.0)	1591 (37.1)	209 (4.9)		41414 (99.3)	32 (0.7)	
Yes	2380 (54.4)	1725 (39.5)	268 (6.1)	0.001	44311 (96.9)	161 (3.5)	<0.001

N; Number, %; percentage, BCG; bacillus Calmette–Guérin
Percentages may not add to 100 due to rounding
*Chi-square test P-value

Table S3 Relationship between participant characteristic and each of following social factor (smoking, substance misuse, prison stay)

Characteristics	Ever smoked			Ever been homeless			Ever been in prison		
	No	Yes	P-value	No	Yes	P-value	No	Yes	P-value
	N (%)	N (%)		N (%)	N (%)		N (%)	N (%)	
Area-deprivation									
Most-deprived	3901 (79.7)	991 (20.3)		4798 (97.9)	102 (2.1)		4820 (98.5)	75 (1.5)	
Moderately-deprived	2749 (82.3)	591 (17.7)		3283 (98.2)	61 (1.8)		3303 (98.7)	43 (1.3)	
Least-deprived	401 (83.5)	79 (16.5)	0.005	477 (99.6)	2 (0.4)	0.036	476 (99.6)	2 (0.4)	0.116
Ever smoked									
No				7280 (98.6)	104 (1.4)		7328 (99.3)	55 (0.7)	
Yes				1659 (95.7)	74 (4.3)	<0.001	1660 (96.0)	70 (4.1)	<0.001
History of substance misuse									
No	7351 (82.4)	1569 (17.6)		8779 (98.3)	155 (1.7)		8835 (99.0)	94 (1.1)	
Yes	33 (16.7)	160 (83.3)	<0.001	169 (87.6)	24 (12.4)	<0.001	163 (84.5)	30 (15.5)	<0.005
Ever been homeless									
No	7280 (81.4)	1659 (18.6)					8859 (99.0)	91 (1.0)	
Yes	104 (58.4)	74 (41.6)	<0.005				144 (81.4)	33 (18.6)	<0.005
Ever been in prison									
No	7328 (81.5)	1660 (18.5)		8859 (98.4)	144 (1.6)				
Yes	55 (44.0)	70 (56.0)	<0.005	91 (73.4)	33 (26.6)	<0.001			
Gender									
Male	3286 (72.4)	1255 (27.6)		4431 (97.6)	111 (2.4)		4444 (97.9)	97 (2.1)	
Female	4063 (89.6)	473 (10.4)	<0.005	4479 (98.5)	68 (1.5)	0.001	4515 (99.4)	28 (0.6)	<0.001
Place of birth									

Non- United Kingdom	6395 (83.6)	1249 (16.23)		7511 (98.2)	139 (1.8)		7569 (99.0)	75 (1.0)	
United Kingdom	979 (67.1)	481 (33.0)	<0.005	1426 (97.3)	39 (2.7)	0.032	1416 (96.6)	50 (3.4)	<0.005
Ethnicity									
Asian	4589 (86.3)	730 (13.7)		5270 (99.1)	49 (0.9)		5285 (99.4)	33 (0.6)	
White	634 (57.5)	468 (43.5)		1077 (96.9)	34 (3.1)		1080 (97.4)	29 (2.6)	
Black	1081 (82.8)	224 (17.2)		1242 (95.0)	65 (5.0)		1277 (97.8)	29 (2.2)	
Mixed/Other	904 (76.7)	274 (23.3)	<0.001	1150 (97.6)	28 (2.4)	<0.001	1145 (97.2)	33 (2.8)	<0.001
HIV status									
No	6855 (81.0)	1605 (19.0)		8310 (98.1)	164 (1.9)		8352 (98.6)	119 (1.4)	
Yes	38 (74.5)	13 (25.5)	0.237	45 (88.2)	6 (11.8)	<0.005	48 (94.1)	3 (5.9)	0.007
BCG vaccination									
No	1138 (80.9)	269 (19.1)		1381 (97.9)	29 (2.1)		1387 (98.4)	22 (1.6)	
Yes	5106 (80.7)	1221 (19.3)	0.877	6215 (98.1)	121 (1.9)	0.717	6243 (98.6)	87 (1.4)	0.590
Recent TB contact									
No	3832 (86.1)	618 (13.9)		4378 (98.0)	73 (1.6)		4414 (99.2)	34 (0.8)	
Yes	3485 (76.1)	1096 (23.9)	<0.001	14489 (97.8)	103 (2.2)	0.038	4498 (98.0)	92 (2.0)	<0.001

N; Number, %; percentage
Percentages may not add to 100 due to rounding
*Chi-square test P-value
